# A Secure Routing Protocol for Wireless Sensor Networks Considering Secure Data Aggregation

**DOI:** 10.3390/s150715127

**Published:** 2015-06-26

**Authors:** Triana Mugia Rahayu, Sang-Gon Lee, Hoon-Jae Lee

**Affiliations:** Department of Ubiquitous IT, Dongseo University, Busan 617-716, South Korea; E-Mails: gia.sutriadi@gmail.com (T.M.R.); hjlee@dongseo.ac.kr (H.-J.L.)

**Keywords:** wireless sensor network, security, energy-efficient, secure routing protocol, secure data aggregation, LEACH, ESPDA

## Abstract

The commonly unattended and hostile deployments of WSNs and their resource-constrained sensor devices have led to an increasing demand for secure energy-efficient protocols. Routing and data aggregation receive the most attention since they are among the daily network routines. With the awareness of such demand, we found that so far there has been no work that lays out a secure routing protocol as the foundation for a secure data aggregation protocol. We argue that the secure routing role would be rendered useless if the data aggregation scheme built on it is not secure. Conversely, the secure data aggregation protocol needs a secure underlying routing protocol as its foundation in order to be effectively optimal. As an attempt for the solution, we devise an energy-aware protocol based on LEACH and ESPDA that combines secure routing protocol and secure data aggregation protocol. We then evaluate its security effectiveness and its energy-efficiency aspects, knowing that there are always trade-off between both.

## 1. Introduction

A Wireless Sensor Network (WSN) is a type of wireless *ad hoc* network that deploys a large number of low-cost sensor devices distributed over an area of interest. Collaboratively, they report sensor readings to a data collection sink or Base Station (BS), regularly or based on demand. The potential uses of this network range from military to civil applications. Their data can be as simple as measurements of physical parameters, such as temperature, pressure, relative humidity, *etc*, to as complex as multimedia content, as in recent years we have seen the exploration of wireless video/visual sensor networks for a wide range of applications [1,2].

Beside their memory capacity limitations, these low-cost sensors are limited in computation, communication capability and are generally battery-powered devices. Therefore, such resource-constrained devices require energy-aware protocols. Furthermore, WSNs deployments are commonly characterized by hostile and unattended environments, and since low-cost sensors are not tamper-resistant devices, consequently this type of network is vulnerable to a variety of attacks. Therefore, WSNs not only need energy-aware protocols, but also the secure ones [3].

As WSNs are deployed to sense and collect information from certain areas of interest for particular applications, secure routing protocols are of important parts since they help govern how those sensor readings can be securely delivered to the BS. A number of secure routing protocols have been proposed [4].

Data communication in WSNs constitutes a great share of the network energy consumption. A Sensor Network Encryption Protocol (SNEP) simulation shows that data communication consumed 71% of the energy cost of computation and communication. SNEP is one of the base components of a set of Security Protocols for Sensor Networks (SPINS) [5], one of the secure routing protocols for WSNs. Therefore in-network process/data aggregation really relieves network burden since it reduces the amount of data that travels to the BS and enforces short range communication for a large amount of sensor nodes, thus conserving network energy. Many secure data aggregation protocols have been proposed as well [6,7].

In this field, our interest is in the combinations of secure data aggregation protocols and secure routing protocols. It is very important to provide a secure routing protocol in order to optimize the security of any data aggregation protocol. The remainder of this article is organized as follows: Section 2 presents the related works. A literature review is described in Section 3. Section 4 includes our security analysis on the reviewed protocols. Our secure routing protocol that considers secure data aggregation is described in Section 5, followed by evaluation of the proposed protocol in Section 6 and finally the conclusions in Section 7.

## 2. Related Works

To the best of our knowledge, only the works of Wu *et al.* [8] and SELDA [9] incorporate secure routing into the secure data aggregation schemes. They employ distributed monitoring schemes to detect misbehaving nodes. When any compromised node is detected by its neighborhood, a secure routing protocol is launched to choose safe path(s) to the considered honest data aggregators. Their secure routing protocols are an on-demand or reactive protocol type. Their main advantage is that, as long as no intrusion is detected, the expensive secure data transmission is not needed. However, it is very obvious that their monitoring scheme requires low-cost sensor nodes to keep watching their neighborhood. Keep sensor nodes awaking all the time consumes a lot of energy.

Different from previous works, proactively providing a secure routing protocol as the foundation for secure data aggregation can be another consideration. This approach makes a secure routing algorithm available since the beginning of network operation and the secure data aggregation scheme can work on top of it. It protects the transmission for the secure data aggregation scheme. We found that there has been no work that lays a secure routing protocol as the foundation for a secure data aggregation protocol.

Due to its energy-efficient feature, we exclusively studied a clustering-based routing protocol, the Low-Energy Adaptive Clustering Hierarchy (LEACH) protocol [10]. Our previous work on LEACH-based secure routing protocols in [11] revealed an interesting fact. Every scheme still trusts the CHs to do the data aggregation. Since the network setting is a homogenous network, this approach is risky.

If we look carefully at the secure data aggregation protocols presented in [6,7], those schemes provide techniques (such as interactive proof, witness-based scheme, reference-based data aggregation, verification or privacy homomorphic encryption scheme) to verify the correctness of the aggregated data or not to fully authorized the aggregators to do the data fusion. This teaches us not to trust the aggregators blindly. Secure data aggregation for a low-cost homogeneous WSN is highly challenging.

We also found that those secure data aggregation protocols are not built on a secure routing protocol. In [12], we stated our premise that although some assumptions are made, clearly defining the secure routing protocol for the secure data aggregation to be based on, or at least stating the minimum requirements for the secure routing schemes to meet in the case for flexible choice of secure routing, is necessary. Therefore we argue that the role of secure routing would be rendered useless if the data aggregation scheme built on it is not secure (i.e. by fully trusting data aggregators). Conversely, the secure data aggregation protocol needs a robust underlying routing protocol as its foundation in order to be effectively optimal.

## 3. Literature Reviews

### 3.1. WSN Security Requirements

In order to devise a security protocol we need to recognize the information security objectives of the protocol and then put a careful effort into adequately addressing those security requirements of each aspect of the protocol. In general there are four information security objectives from which many others will be derived. They are confidentiality, data integrity, authentication and non-repudiation. In [6] Ozdemir *et al.* explained the required security properties for WSNs and even related them to the data aggregation process:

- Data confidentiality—To ensure that the content of the message should not be revealed to the unauthorized receiver. Some secure data aggregation schemes provide this property on a hop-by-hop basis in which any aggregator node needs to decrypt the received encrypted data before applying the aggregation function on it and then encrypt the aggregated data before transmitting it to the higher level aggregator or directly to the BS, while the other schemes provide end-to-end data confidentiality in which any aggregator node directly applies the aggregation function to the received encrypted data.
- Data integrity and freshness—Data integrity guarantees that the message has not been altered during the propagation. If data aggregation is employed, then it is not possible to have end-to-end data integrity since data aggregation causes alterations. Data freshness protects data aggregation from reply attacks.
- Source authentication—Enables sensor nodes to ensure the identity of the peer node that they are communicating with. A compromised node can launch a Sybil attack in which it may send data under several fake identities in order to corrupt the aggregated data.
- Availability—To guarantee the survivability of network services against DoS attacks. The attack aiming at an aggregator can make some part of the network lose its availability because the aggregator is responsible for providing the measurement of that part of the network.

### 3.2. LEACH

**Figure 1:**
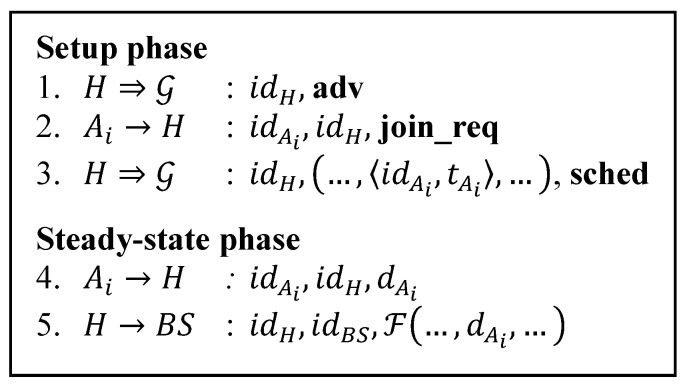
LEACH protocol.

There are major routing protocols for WSN such as clustering-based routing protocols, TinyOS beaconing, directed diffusion, geographic routing, minimum cost forwarding, rumor routing and energy conserving topology maintenance [4]. Clustering-based routing is famous for its energy-efficient features. One of protocols in this family is LEACH. LEACH rotates randomly the role of CHs in order to evenly distribute the energy load among the sensors in the network. It incorporates a data aggregation scheme into its routing protocol to reduce the amount of information that CHs should transmit to the BS. LEACH considers a network model where a fixed BS is located far from sensors and all sensors in the network are homogeneous and energy-constrained. LEACH assumes that every node can reach the BS by transmitting with sufficiently high-power. Figure 1 summarizes LEACH protocol in steps.

The various symbols denote:

$H,A_{i},\;BS$:   A CH, ordinary node, and BS, respectively;

G: The set of all nodes in the network;

⇒,→: Broadcast and unicast transmissions, respectively;

$id_{X}$:   Id of node X;

**adv**, **join_req**, **sched**: String identifiers for message types;

$<id_{X},\;t_{X}>$: A node id X and its time slot $t_{X}$ in its cluster’s TDMA schedule;

$d_{X}$: Sensed data from node X;

ℱ( ): Data aggregation function.

LEACH divides network operation processes into rounds. Every round consists of two phases, *setup phase* and *steady-state phase*. Cluster formation is done in the setup phase. This phase is comprised of three steps. At first, the self-elected CH candidates advertise the **adv** message to become CHs for that round. In the next step, based on the received signal strength of the **adv** message, nodes send the **join_req** message to a CH they want to join. In the last step CHs send the **sched** messages containing TDMA time slots to their cluster members. After cluster formation, LEACH enters the steady-state phase. The actual communication takes place in this phase. At first cluster members send their sensor readings to the CH. Then the CH aggregates the readings and sends the aggregated data to the BS.

LEACH only concerns energy efficiency. Although its randomized rotation of CHs’ roles inherently provides protection for the aggregator nodes, LEACH does not take the security aspect into consideration. Thus several schemes, such as SLEACH [13], SecLEACH [14], SC-LEACH [15], Armor LEACH [16] and MS-LEACH [17], have been proposed to secure LEACH.

### 3.3. LEACH-Based Secure Routing Protocols

Since SC-LEACH lacks a clear detailed description for its security scheme, no further discussion is provided for it. No discussion for Armor-LEACH is provided either since its security protocol is basically the same as SecLEACH.

#### 3.3.1. SLEACH

**Figure 2:**
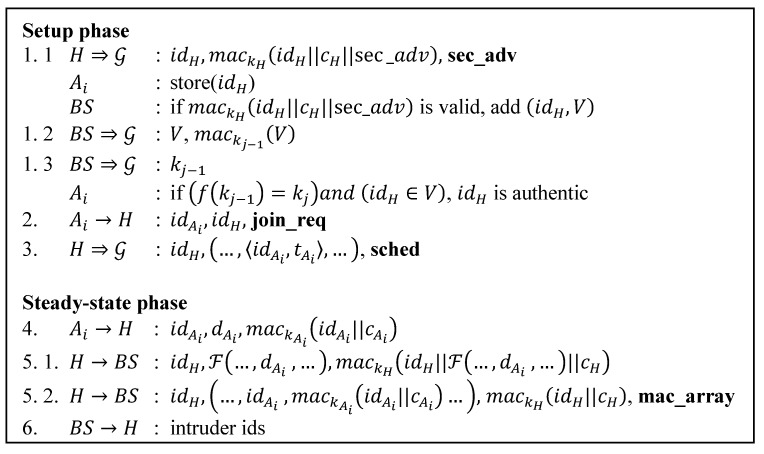
SLEACH protocol.

SLEACH is the first protocol that attempts to add security to LEACH. Figure 2 summarizes this protocol.

Additional symbols denote:

$k_{X}$: Symmetric key shared by node X and BS;

$mac_{k}\left(\;\right)$: MAC calculated using key k;

||: Concatenation operator;

**sec_adv**, **mac_array**: String identifier for message type;

V: An array of node ids, the legitimate CHs list;

$c_{X}$: Counter shared by node X and BS.

Prior to deployment each sensor node *X* is loaded with two keys: key *X_X_*, a master symmetric key that node X shares with the BS; and key $k_{j},$ a group key that is shared by all members in the network. From $X_{X}$, the key holders derive $k_{X}$ for message authentication code (MAC) computation and verification. $k_{j}$ is the last key of a key sequence S generated by applying a one-way hash function f recursively to an initial key $k_{0}$ ($S=k_{0},k_{1},\ldots ,k_{j-1},k_{j}$ where $f\left(k_{j-1}\right)=k_{j}$ ). At the beginning BS keeps S secret, but then it will reveal the last element $k_{j}$ to the network periodically. SLEACH uses *μ*TESLA to do broadcast authentication.

In the first step of the setup phase, CH candidates broadcast a modified advertisement **sec_adv** message to the network. This message contains the CH’s id and the MAC of the CH’s id, **sec_adv** and its counterpart. CH calculates the MAC using the $k_{H}$ shared with BS. Upon receiving advertisement messages, each node stores the ids of CHs. On the BS side, when it receives any advertisement message, it verifies the MAC of the message. If the message is valid, the BS puts the corresponding CH’s id into the list V of legitimate CHs.

After compiling the list V, BS broadcasts the list along with its MAC computed with $k_{j-1}$. BS then reveals $k_{j-1}$ after some time. Every node then verifies the validity of $k_{j-1}$ ($f\left(k_{j-1}\right)$ should equal $k_{j}$) and checks if the id of the chosen CH candidate is in the list of the legitimate CHs.

Then in the second step, each node sends its join request **join_req** to a legitimate CH in which it wants to join. In the last step of the setup phase, CHs broadcast a schedule message **sched** containing TDMA time slots to their cluster members.

Having done the cluster formation, network enters the steady-state phase. This starts with a cluster member sending its sensor reading to its CH according to the time slot assigned for it. The message is shown in step 4. It contains the sender’s id, its sensor reading, and the MAC of its id and the counter calculated using the key shared with the BS.

After collecting the data, CH then aggregatesthem. It creates a message containing its id, aggregated data and the MAC of them both. It also includes its counter shared with the BS in the MAC computation. Then it sends this message to the BS. This is shown in step 5.1.

Another message from a CH to BS is the MAC array of its cluster members **mac_array**. Step 5.2 shows this message. MACs pairs are taken from the message in step 4.

In the final step BS verifies every MAC of the sensor nodes. If any MAC is invalid, BS drops the whole packet and sends back the list of illegitimate sensors to the corresponding CHs, so they can blacklist them for the next round.

#### 3.3.2. SecLEACH

**Figure 3:**
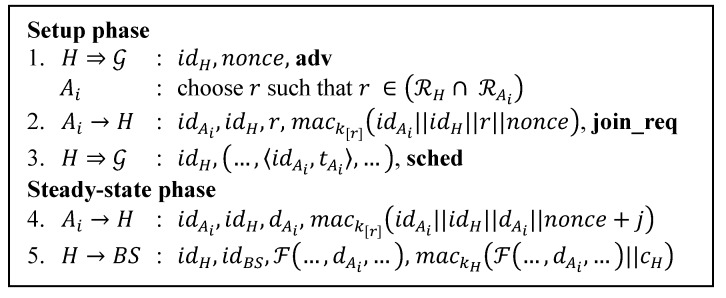
SecLEACH protocol.

SecLEACH was proposed to enhance SLEACH. Figure 3 summarizes the SecLEACH protocol. It uses the random key pre-distribution scheme proposed by Eschenauer and Gligor [18]. Prior to deployment each node is given a secret key (shared only with BS) and a set of key rings drawn from a large key pool. Those key rings contain pairs of key ids and keys.

In the setup phase, every self-elected CH broadcasts its advertisement message **adv** containing its id and a nonce. After receiving all advertisement messages, a node will choose a CH that it wants to join based on the signal strength and choose the index of secret common keys that it shares with the chosen CH.

Additional symbols denote:

r: Key index in the key rings;

$R_{X}$: The node X’s key ring;

$k_{\left[r\right]}$: Symmetric key associated with r;

j:   Reporting cycle within current round.

In step 2, a node sends its join request message **join_req** to a CH containing its id, CH’s id, the index of shared keys and also the MAC calculated using the shared key. The advertisement message’s nonce is included in the MAC computation. After that the CH broadcasts the schedule message **sched** to its cluster members.

In steady-state phase, each cluster member sends its sensor readings to CH on its time slots. As shown in the fourth step, SecLEACH includes sensor readings in the MAC computation. It uses the key it shares with the CH to compute MAC. It also puts nonce and the reporting cycle of the current round into the MAC computation to provide freshness. In step 5, the CH sends the aggregated data to BS along with the MAC calculated using the key shared with BS. It also puts the counter shared with BS into the MAC computation.

#### 3.3.3. MS-LEACH

**Figure 4:**
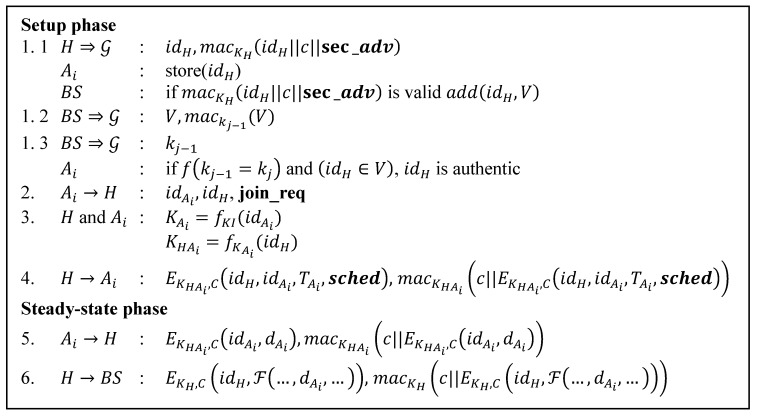
MS-LEACH protocol.

MS-LEACH was proposed to enhance the security of SLEACH by providing data confidentiality and node-to-CH authentication using pairwise keys shared among nodes. Figure 4 shows the protocol.

Step 1 and 2 are similar to SLEACH. In step 3, pairwise keys are generated between the CH and its member nodes. In step 4, the CH unicasts a TDMA schedule **sched** encrypted with the pairwise key and a counter to each member along with the MAC of the counter and the encrypted form of the TDMA schedule calculated with the pairwise key sent.

In steady-state phase, every member sends the encrypted form of its measurement data and id calculated using the pairwise key and counter value to the CH along with the MAC value of the counter and the encrypted form of the node’s id and its measurement data calculated also using the pairwise key. In step 6, the CH encrypts its id and aggregated data using the key $K_{H}$ and counter shared with BS and also compute MAC of counter and the encrypted message using $K_{H}$. Then it sends the encrypted massage and MAC to BS.

Additional symbols denote:

c:   Counter;

KI: The symmetric last key chain held by BS preloaded in each node;

$f_{K}$: A family of pseudo-random function.

### 3.4. ESPDA as a Secure Data Aggregation Protocol

There are many secure data aggregation protocols proposed so far. The works in [6,7] build many approaches to classify them. For example, the classifications are based on adversary types, single/multiple aggregator model, the existence of verification phase, the types of attacks faced, the security properties provided, the cryptographic primitives used, and the type of sensor data (plain or encrypted sensor data). However, for briefness, we only explain a protocol that is used in our proposed protocol.

**Figure 5:**
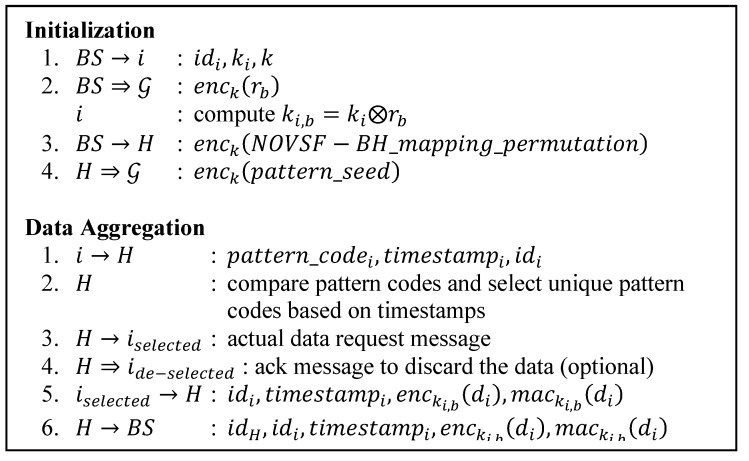
ESPDA protocol.

Where the various symbols denote:

i,H,BS: A sensor node i, a CH and BS, respectively;

⇒,→: Broadcast and unicast transmissions, respectively;

$id_{i}$: The id of node i;

$d_{i}$: Sensed data from node i;

$pattern\_code_{i},timestamp_{i}$: The pattern code and timestamp of node i;

$k_{i},r_{b},k_{i,b}$: The secret built-in key of node i, the random session number from BS and node specific session key of node i, respectively;

$mac_{k}\left(\;\right)$: MAC calculated using k key;

$i_{selected}$, $i_{de-selected}$: The selected set of node that has unique pattern codes and unselected ones, respectively.

Energy-efficient and Secure Pattern based Data Aggregation (ESPDA) [19] works on clustered-based WSNs and is suitable for LEACH-based secure routing protocols. Figure 5 shows a summary of ESPDA protocol. The main idea of ESPDA is that instead of directly sending the actual sensed data to the CH, the sensor nodes send their pattern codes first. The pattern code is data representative of the sensed data and is generated based on the secret pattern seed which is periodically distributed by the CH. The CH then compares those pattern codes, selects only the unique ones and requests the actual data from the nodes having the corresponding unique codes. In reply, each selected node then sends the encrypted actual data to the CH while the rest may be instructed to drop the data. The CH does not need to decrypt the received encrypted data because the data aggregation process is done prior to the actual data transmission. This reduces the overhead of the CH and thus contributes to the energy efficiency. Then CH can forward the message to the BS.

Each sensor node is assigned a unique id ($id_{i}$), a node specific secret key ($k_{i}$) and a secret key common to all nodes (k) prior to the deployment by the BS. In addition, BS periodically broadcasts a random session number ($r_{b}$) in encrypted form using key k which is $enc_{k}\left(r_{b}\right)$. Upon receiving $r_{b}$, any node i computes the node specific session key ($k_{i,b}$) for data communication by XOR-ing its built-in secret key $k_{i}$ with $r_{b}$, $k_{i,b}=k_{i}⊗r_{b}$. Later on node i uses $k_{i,b}$ to encrypt its actual data. Accompanying this encrypted data, node i also sends its timestamp and id. Those two pieces of data will help the BS to choose the right $r_{b}$ and compute the right $k_{i,b}$ to decrypt the message. In order to provide data integrity, the MAC of the message computed with $k_{i,b}$ is also included in the message.

This protocol uses the Non-blocking OVSF Block-Hopping (NOVSF-BH) technique. This technique improves the security and the spectral efficiency of the network. In NOVSF codes, each OVSF code has 64 time slots such that any number of this timeslots can be assigned to a channel. The proposed NOVSF-BH technique assigns data blocks to time slots using different mapping in every session, so besides equipping every sensor node with keys, the BS also periodically sends a different mapping permutation in encrypted format using the key k ($enc_{k}\left(permutation\right)$) to CHs. This mapping permutation allows every node to map its data blocks according to the given code before sending it to the CH. By doing so, any intruder first has to find the mapping pattern for that particular session and then try to decrypt the message.

Figure 5 depicts how the protocol runs in one session. In every new session the BS broadcasts a new $r_{b}$. It encrypts $r_{b}$ using key k. The BS also sends the mapping permutations encrypted with key k to CHs. Reference [19] does not explain how CHs will distribute this mapping permutation within the cluster, but because all legal nodes in the network share the network key k, this task is trivial. Each CH may broadcast it within its own cluster while providing the confidentiality, authenticity and integrity with the shared common key k. Also each CH broadcasts the pattern seed encrypted within its cluster.

### 3.5. Identity-Based Cryptography

For efficient communication, identity-based cryptography [20,21] will be adapted for our secure routing protocol design. Identity-based cryptography is a type of public-key cryptography in which a publicly known string representing an individual or organization is used to derive a public key. In a WSN setting, a node’sid, which is publicly known, is used to derive its public key. In non-interactive key agreement scheme, any two nodes use other node’s id and derive a mutual secret without even communicating. This mutual secret, unknown to any other nodes, can be used to generate a cryptographic key for secure communication between the nodes under consideration. Though it still suffers from the high-computation cost of pairing, it is promising because there has good research outcomes for sensor network applications [22].

#### 3.5.1. Identity-Based Non-Interactive Key Agreement

Sakai *et al.* [23] proposed an identity-based non-interactive key agreement scheme based on bilinear pairings. This scheme is used to bootstrap the security in WSNs. Each node gets a unique id and a secret that is not shared with any other node. Two such nodes use other node’s id and derive a mutual secret without even communicating. This mutual secret, unknown to any other nodes, can be used to generate a cryptographic key for secure communication between the nodes under consideration. Such a scheme is referred as identity-based non-interactive key agreement scheme. Because of its non-interactivity, this approach is very suitable for establishing secret keys among resource-constrained nodes.

#### 3.5.2. Identity-Based Digital Signature

For the authentication of broadcast messages in the proposed protocol, we will use an online/offline identity-based digital signature which is suitable for low-cost wireless sensor networks. The online/offline identity-based digital signature performs the signature generation procedure in two phases. The first phase is performed offline (prior to the knowledge of the message to be signed) and the second phase is performed online (after knowing the message to be signed). The online phase is typically very fast, and hence can be executed efficiently by sensor nodes. One efficient online/offline identity-based signature solution for the proposed protocol can be found in [24]. The contributions of that protocol are:

- It does not require any certificate attached to the signature for verification.
- It does not require any pairing operation in signature generation or in verification.
- It allows the offline information to be re-usable. The signer is not required to execute the offline algorithm every time it wants to sign a new message. Furthermore it does not require any secret information from the signer. Thus it saves a lot of communication bandwidth since the node does not need to return to the BS for the renewal of the offline information every time signing is performed.

## 4. Security Analysis of LEACH-Based Secure Routing Protocols and ESPDA

### 4.1. Security Analysis of LEACH-Based Secure Routing Protocols

LEACH-based secure routing protocols consider CHs as trusted entities which is undesirable from a data fusion point of view. This fact triggers insight into our security protocol requirements when we analyze the security of each routing protocol.

#### 4.1.1. Security Analysis of SLEACH

In SLEACH, nodes are not equipped with secret keys to secure the communication among them. Some main drawbacks have their root in this absence. The following are the observed drawbacks of this protocol:

- Non-authenticated join request messages allow intruders to join the network freely. Cluster member verification can only be done at the BS level, not at the CHs level. This situation can lead to wasted network resources since any aggregated data containing data from an illegitimate member will be dropped by the BS.
- Intruders can exploit the plain-text non-authenticated schedule messages to disrupt the communication. For example by sending false schedules, data transmission collisions may happen. Therefore schedule messages need authentication and integrity protection.
- In the case of applications where the data are sensitive information, the transmission of plain-text sensor readings is not desirable, and they need confidentiality protection. Another problem with the sensor readings transmission happens also to the aggregated data transmission. The BS cannot afford authentication for aggregated data though it can verify cluster member authentication using the accompanying MAC.
- No key update provisioning for key $k_{j}$.
- Not knowing the previous blacklisted nodes, the new CHs will include those old intruders in the data gathering process. This may lead to more aggregated data get dropped before the new list of intruders gets issued by the BS.

#### 4.1.2. Security Analysis of SecLEACH

By deploying a random key pre-distribution for node-to-CH authentication, SecLEACH improves upon SLEACH. It thus cuts down the steps where the BS needs to verify each cluster members before accepting the aggregated data sent by their CHs.

The join request message is now authenticated, therefore it prevents intruders from joining the network. Different from SLEACH, SecLEACH provides data authentication from member nodes to CHs by including sensed data in the MAC computation. This scheme helps the BS to verify the data integrity. However, SecLEACH still inherits some of SLEACH’s drawbacks. Those are:

- The data integrity of schedule message is not provided. The problem found in the SLEACH protocol applies here.
- The sensed data is not encrypted, this scheme lacks of confidentiality property in the case the data is sensitive.

#### 4.1.3. Security Analysis of MS-LEACH

**Figure 6:**
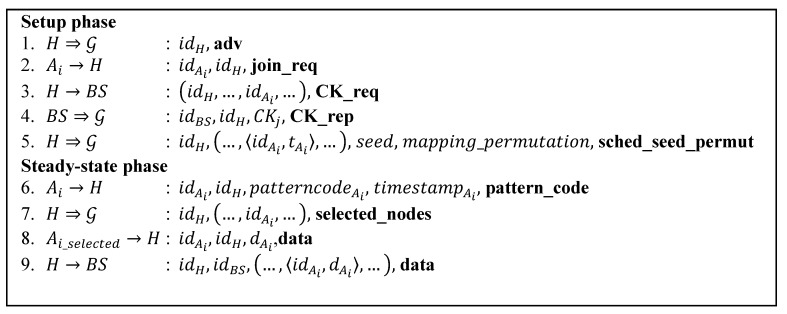
Message flow of the proposed protocol.

Below are the observed drawbacks of the MS-LEACH protocol:

- Proper counter management is not provided. The counter c in the step 1.1 and 6 should be different from the one in steps 4 and 5 (readers should refer to Figure 6 for the MS-LEACH protocol).
- MS-LEACH does not provide authentication for join request messages.
- The non-authenticated join request messages invite intruders to send them to the CH freely. For this reason, even though the protocol provides confidentiality, integrity and reply attack protection for the **sched** message after the attack has been launched, an attacker can still disrupt the timeslot schedule. The CH cannot remove the timeslot already assigned to it or even to rebuild the timeslot. As found in SLEACH, there is no key update provisioning for key $k_{j}$.
- Multiple unicasts for schedule messages can deplete the energy of a CH.

These three protocols still trust CHs to do the data aggregation. Since BS is the only trusted party in the network, these schemes still lack secure data aggregation.

### 4.2. Security Analysis of ESPDA

The drawbacks of ESPDA protocol are explained in the following points:

- The use of a static network key k is risky. If any intruder is able to successfully obtain knowledge about the key k, then security of the protocol can be destroyed. Therefore rekeying for the network key k is needed to mitigate this problem.
- Because the static network key k is used to encrypt the $r_{b}$, pattern seed and mapping permutations, ESPDA does not provide freshness for this information. It is not clear how the source authentication and message integrity are provided for ESPDA. Even though we assume the id of the sender is included in the message and MAC computation so that the message origination and integrity service are available to the receiver, this protocol is still vulnerable to replay attacks. Any intruder may rebroadcast the previously captured messages by distributing $r_{b},$ pattern seed and mapping permutations in another different session. The nodes having different $k_{i,b}$ with that of the BS will fail in the BS decryption process. Some nodes may have different pattern seeds than the rest of the cluster which results in inaccurate data aggregation. The CH is not able to reconstruct the proper order of data blocks because different mapping permutations were used by the member nodes. Therefore a dynamic session-dependent group key can be a possible solution for this drawback.
- The data integrity of pattern code messages from sensor nodes to the CH is not provided. Any intruder can alter the pattern code message and disrupt the data aggregation. The integrity of the message from sensor nodes cannot be verified at the CH but only at the BS. No provisioning of the early invalid data detection in the BS causes energy consumption in vain. A network key k can be used to provide data integrity for the message.
- The source authentication only exists between nodes and the BS, not between nodes and the CH. Any intruder may launch impersonation attacks in which any unauthenticated node may impersonate any legal node. Though the intruder does not have the legal secret key shared with the BS, it can send its pattern code to a CH. The CH runs the pattern comparison algorithm with the received pattern code and requests the actual data from that illegal node if its pattern code is unique. Because an illegal data insertion cannot be detected by the CH and reaches BS, it consumes network resources. Providing shared secret among nodes may be a possible solution.
- In the protocol, the CH requests their actual data from the selected nodes for multi-unicast communication. This can be expensive. One broadcast message with an authentication service may be a solution.

## 5. Proposed Protocol

### 5.1. Message Flow of the Proposed Protocol

Figure 6 shows the proposed protocol’s message flow. It involves eight message types. They are the **adv**, the **join_req**, the **CK_req**, the **CK_rep**, the **sched_seed_permut**, the **pattern_code**, the **selected_nodes** and the **data** messages. This message flow is presented without any cryptographic function being involved.

The various symbols denote:

H,$\;A_{i}$, BS: A CH, an ordinary node, and BS, respectively;

G: The set of all nodes in the network;

⇒,→: Broadcast and unicast transmissions, respectively;

$id_{X}$: The ID of node X;

**adv**, **join req**, **CK_req**, **CK_rep**, **sched_seed_permut**, **pattern_code**, **selected_nodes**, **data**, **sched**: String identifiers for message types;

$<id_{X},\;t_{X}>$: A node id X and its time slot $t_{X}$ in its cluster’s TDMA schedule;

$d_{X}$: Sensor measurement of node X;

mapping_permutation: NOVSH-BH mapping permutation;

seed: Pattern seed;

$pattern\_code_{X},$ $timestamp_{X}$: The pattern code and timestamp of node X;

$X_{selected}$, $X_{de-selected}$:   The selected set of node that has unique pattern codes and unselected ones, respectively.

Like the LEACH protocol, the proposed protocol works in rounds. Every round has two phases, the setup phase and the steady-state phase. Setup phase is the phase where the cluster formation is done. At the beginning of that phase, every node that wants to become the CH in the coming round broadcasts an advertisement message, the **adv**, containing its id. Upon receiving an **adv** message, a node that wants to join the cluster sends back the join request message, **join_req**, to the corresponding CH. Having listed all joining nodes, a CH then sends a request, the **CK_req** message, to the BS asking for a cluster key for its cluster. In reply, the BS broadcasts the **CK_rep** message containing the cluster key and the id of the CH so that the nodes that belong to that CH can take this message. After that, the CH broadcasts the **sched_seed_permut** message within its own cluster. The message includes the TDMA schedule, the pattern seed and the mapping permutation.

In the steady-state phase, at first every node in a cluster, according its timeslot, sends a **pattern_code** message to the CH. The **pattern_code** message contains the ids of the sending node and the CH, the representative data of sensor reading and the timestamp of the sensor reading. After collecting all the pattern codes of its cluster, the CH then compares those pattern codes and selects the unique ones. Later on the CH broadcasts the **selected_nodes** message containing the list of nodes having the unique pattern codes to its cluster. Then the selected nodes send the **data** message to the CH. The **data** message contains the ids of the sending node and the CH and the actual sensor reading. Finally, the CH forwards all the collected data to the BS as the **data** message.

As we can see from steps 3 and 5, there are two types of message which are broadcasted within a certain cluster, the **sched_seed_permut** and the **selected_nodes** message. These broadcast messages need a group key or cluster key to secure them. Therefore we introduce two messages, the cluster key request **CK_req** and the cluster key reply **CK_rep**.

### 5.2. Security Requirements

In order to fulfill these security requirements of WSNs, we consider data integrity, source authentication, data confidentiality and data freshness in applying cryptographic algorithms to the proposed protocol. As shown in Figure 6, eight messages are involved in our protocol. Evaluating the security requirement of each message is vital in equipping every involved party with adequate tools to endorse a secure and robust yet efficient protocol. Table 1 summarizes the security requirements of each message.

The first message, **adv**, requires data integrity protection to prevent any intruder from altering the identity of the announcing node. The impact can be that some nodes try to join a non-existent cluster. At the end of the setup phase those nodes may end up without a CH (orphans). In order to gain network information, it is possible for intruders to advertise themselves as a CH. Therefore a source authentication feature is needed as well. Data freshness is also required in order to avoid reply attacks. The LEACH protocol cannot detect if a particular node has sent an **adv** message twice or more in a round. An intruder can rebroadcast the **adv** messages and some nodes may join non-existent clusters. This situation can delay and disrupt the cluster formation stage.

**Table 1:** Security requirements of the proposed protocol.

Message	Security Requirements				Type of Message	Remark
	Data Integrity	Source Authentication	Data Confidentiality	Data Freshness		
Advertisement, **adv**	☑	☑		☑	Broadcast	
Join request, **join_req**	☑	☑		☑	Unicast	
The schedule, pattern seed and mapping permutation, **sched_seed_permut**	☑	☑	☑ Except for schedule	☑	Broadcast	
Pattern code, **pattern_code**	☑	☑		☑	Unicast	
Notification for the selected nodes, **selected_nodes**	☑	☑		☑	Broadcast	
Sensor readings, **data**	☑	☑	☑	☑	Unicast	
Cluster key request, **CK_req**	☑	☑		☑	Unicast	Ours
Cluster key reply, **CK_rep**	☑	☑	☑	☑	Broadcast	Ours

The join request message, the **join_req**, requires source authentication in order to prevent illegitimate nodes from joining the network. Malicious cluster members can send fake sensor readings to give inaccurate network information. Crowding the timeslots of the TDMA schedule can be another option for intruders by sending many fake **join_req** messages. It can delay the actual sensor data gathering. Therefore source authentication is important. Data integrity is also required so that unwanted alterations can be detected. The impact if the id of the sender is changed to another node’s id is that the CH will incorrectly recognize its cluster members and later on falsely assign the timeslot to the wrong node. If the id of the CH is changed, then the joining node may falsely join another cluster or ends up as an orphan node. In dealing with replay attacks which takes place at later rounds, data freshness is required.

Except for the schedule part that does not need data confidentiality, the **sched_seed_permut** message needs data integrity, source authentication, data confidentiality and data freshness. This message contains the TDMA schedule for a cluster, pattern seeds for the member nodes to generate lookup tables and the mapping permutation of the NOVSF-BH technique. Their security requirements are detailed as follows.

The communication within a cluster can be disrupted just by changing the schedule for the cluster members. Therefore the schedule needs data integrity protection. Without source authentication, it cannot be guaranteed that the schedule comes from an authorized CH. Anyone can send fake schedules and disrupt the communication within a cluster. As some nodes may join the same CH at the later round, replaying an old schedule may affect those nodes which leads to communication disruption. Because all members in a cluster must listen to the schedule message, no confidentiality protection is needed.

Originating from the ESPDA protocol, the permutation increases the protection of the messages sent by non-CH nodes to CHs. As we know, in ESPDA, before a node sends the sensor data to its CH, it encrypts the data and then assigns the data blocks to the NOVSF time slots based on the mapping permutation. Therefore, at first any intruder needs to find out the proper order of these data blocks, rearrange them properly and then try to decrypt the data in order to recover it. Mapping permutation requires data integrity so that unwanted alterations can be detected. If the CH has a different mapping permutation than the cluster members, then the CH will not be able to reorder the data blocks in the proper way. Data confidentiality is needed so that information breaches can be prevented. If any intruder is able to learn the mapping permutation, then the use of NOVSF-BH has no effect. Source authentication is needed to prevent cluster members from getting false mapping permutations from unauthorized sources. Data freshness is also required to prevent replay attacks. The use of old mapping permutations can prevent CHs from properly reordering the data blocks.

Like mapping permutation, pattern seeds are also from ESPDA. This type of message requires the same security features. Without data integrity, altered pattern seeds may yield different lookup tables that are used to generate the pattern codes within a cluster. Without source authentication, any cluster member can receive a false pattern seed from an unauthorized source that can later disrupt data aggregation. Without data confidentiality, anyone can know the content of the sensitive data. Without data freshness, replay attacks can be launched. The old pattern seeds can result in different lookup tables. As we know the cluster members generate the pattern code based on the lookup table. If different lookup tables exist, two or more of the same sensed data can be represented differently. CHs may notice these pattern codes as unique ones and ask for their corresponding actual data. This may lead to inefficient data aggregation.

Moving on to another message type, the **pattern_code**, this message requires data integrity, source authentication and data freshness in order to detect unwanted alterations, unauthentic sources and replay attacks that can disrupt the proper data aggregation.

In ESPDA protocol, CH requests to the nodes having unique pattern code for the actual sensor readings. These multi-unicast messages can be replaced by one broadcast message, the **selected_nodes** message, but it requires data integrity, source authentication and data freshness in order to avoid unwanted alterations, unauthenticated sources and replay attacks. As in the proposed protocol the steady-state phase is repeated several times in one round, the old **selected_nodes** message can disrupt the data aggregation.

The **data** message which contains sensor readings requires data integrity, source authentication, data confidentiality and data freshness in order to avoid unwanted alterations, unauthorized sources, information breaches and reply attacks.

The CH requests a cluster key from the BS for its cluster via a **CK_req** message. This message contains the list of the cluster members. Thus source authentication feature helps the BS verify the requester’s identity, and the message integrity to detect unwanted alterations. The **CK_req** message yields a broadcast **CK_rep** message in turn, which consumes a lot of network bandwidth. Because anyone can capture and resend this message later at a different round to launch replay attacks, this message requires data freshness.

**CK_rep** is a broadcast message sent by the BS. This message contains the cluster key information. Since the cluster key is secret to a certain group of nodes or clusters, the confidentiality of the message is required in order to prevent cluster key information breaches. The data integrity is also needed to detect any unwanted alteration. Source authentication is required to guarantee BS as an authorized source of the message. Because there is possibility that the same nodes join the same CH at a later different round, replay attacks can cause some members of the cluster to use the old cluster key which then disrupts the communication. Therefore, data freshness is required as well.

### 5.3. Combining LEACH-Based Security Protocol with ESPDA

**Figure 7:**
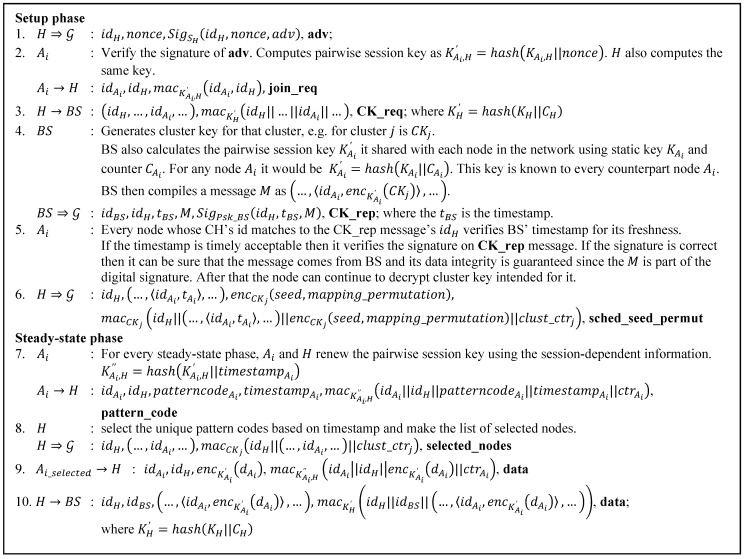
The proposed protocol.

Based on the previous security requirements, a protocol that considers security in the cluster formation, routing mechanism and data aggregation process is proposed. This protocol is summarized by Figure 7. Like the LEACH protocol, the proposed protocol works in rounds and has two phases, the setup phase and steady-state phase.

Before we jump into the explanation on how the proposed protocol addresses those security requirements, the description of counter management in our system is provided first. In order to provide data freshness, we introduce three types of counters. They are counters shared between a node and the BS, a counter that is shared between two nodes in the network and a cluster counter which is shared within a certain cluster. These counters are incremented every time a successful communication takes place between two involved parties whether individual parties or a group. In resetting the counter, the exception is the cluster counter, which is created every time a new cluster is formed and destroyed at the end of a round. The proposed protocol depicted in Figure 7 summarizes how those security requirements are met. The step by step explanation below gives a description of our protocol:

▪ The **adv** message

In order to provide data integrity and source authentication for **adv** messages, we use a digital signature. This approach is different from the SLEACH approach. SLEACH uses MAC to provide data integrity and *μ*TESLA to provide authentication for the CH sending the **adv**. It uses *μ*TESLA with the help of the BS because SLEACH does not have pairwise keys between nodes that can help any node in the network authenticate the CH directly, so the BS, on behalf of any other node in the network, authenticates the CH. It then issues a list of legitimate CHs to the network.

Compared to SLEACH, the proposed protocol’s approach has less communication overhead. In the proposed protocol, every node can directly authenticate a CH after receiving the **adv** message while in SLEACH the process of source authentication is indirect. Every node has to wait for the BS. Furthermore, if SLEACH’s approach is adopted, then we need to provide the key chain management mechanism. Rekeying is needed to update the key chain so the process of *μ*TESLA broadcast authentication can still be maintained. Therefore we avoid adopting the SLEACH approach.

Though SecLEACH does not provide a data integrity feature for the **adv** message, it does provide a source authentication feature. By employing a random key pre-distribution mechanism, SecLEACH cuts down the communication overhead suffered by SLEACH. The use of a shared common key later on verifies the identity of the counterpart which also imposes a source authentication service. Thus we use identity-based key agreement to avoid storing a lot of keys and having orphan nodes in the network. For **adv** message data freshness we use nonce, which ensures freshness for **adv** messages.

Where the various additional symbols denote:

nonce   :   A random number used once;

$Sig_{S_{X}}\left(u,v,\ldots \right)$ : A digital signature computed using private key of node X, the $S_{X}$, and based on inputs (u,v,…);

$K_{X,Y}$, $K_{X}$   :   A mutual secret key of node X and Y, and of node X and the BS, respectively, computed using ID-based non-interactive key agreement scheme;

hash( )   :   A hash function;

$C_{X},cluster\_ctr_{j},ctr_{X}$: A counter node X shared with BS, a cluster key counter of a certain cluster j, and a counter node X shared with its CH, respectively.

▪ The **join_req** message

The data integrity of this message is provided through MAC. The use of a pairwise session key between the CH and the sender gives the message source authentication features. Driving a pairwise session key from a mutual secret key and the nonce of the **adv** message freshens against replay attacks.

▪ The **CK_req** message

The data integrity feature for **CK_req** to protect the list of nodes that are needed for the cluster key is provided via MAC. The use of a secret key shared between the requesting CH and the BS provides source authentication, whereas data freshness is achieved by including the counter that the CH shared with the BS in the computation of the shared secret key.

▪ The **CK_rep** message

Digital signature provides both data integrity and source authentication for the whole message. Data confidentiality is provided via encryption. As the BS encrypts the cluster key for each node listed in **CK_req** message using each corresponding mutual secret key, it also guarantees that the cluster key comes from the authorized BS. The BS also includes counters it shares with each node for the computation of the shared secret key in order to provide data freshness.

▪ The **sched_seed_permut** message

Data integrity is provided using MAC. The use of cluster keys provides source authentication. Because all nodes in the cluster share the cluster key, anyone in that cluster can generate and broadcast a fake **sched_seed_permut** message in order to disrupt the communication and data aggregation. Therefore in order to prevent this from happening, it is very important to authenticate nodes that join a cluster. Data confidentiality of the pattern seed and NOVSF-BH mapping permutation are provided through encryption. A cluster counter is included in the MAC computation to provide data freshness.

▪ The **pattern_code** message

Data integrity is provided via MAC. The use of a mutual secret key between the CH and each cluster member guarantees the source authentication. As **pattern_code** message is the message sent at the beginning of the steady-state phase; we include a timestamp in the computation of the mutual secret key between each node and the CH to mark the beginning this phase. This provides data freshness for every new steady-state phase because our protocol works on multiple steady-state phases. As in every steady state-phase there is more than one communication between clusters and the CH, so we include the counter every node shares with the CH in the MAC computation to provide data freshness.

▪ The **selected_nodes** message

Data integrity is provided via MAC. Source authentication is provided using a cluster key. Like the **sched_seed_permut** message case where anyone having the cluster key can generate and broadcast fake **selected nodes** messages, the prevention depends on the source authentication feature of the **join_req** message.

▪ The **data** message

Data integrity and source authentication are available for communication between cluster members and the CH, and between the CH and the BS via the identity-based key agreement. Data integrity is provided via MAC. The use of a mutual secret key guarantees the source authentication of the message. Data confidentiality features are available via encryption. For the **data** message transmitted within the cluster, its data freshness is provided by including the counter every node shares with the CH in the MAC computation, whereas data freshness for the **data** messages sent by the CH to the BS is provided by including the counter the CH shares with the BS in the mutual session secret key computation. Table 2 summarizes those approaches we use to fulfill the security requirement for the proposed protocol.

**Table 2:** Protocol security requirements fulfillment.

Message	Security Requirements			
	Data Integrity	Source Authentication	Data Confidentiality	Data Freshness
**adv**	Digital signature	Digital signature	-	Nonce
**join_req**	MAC	Mutual key	-	Nonce
**CK_req**	MAC	Mutual key	-	Counter
**CK_rep**	Digital signature	Digital signature	Encryption	Counter, timestamp
**sched_seed_permut**	MAC	Cluster key	Encryption	Counter
**pattern_code**	MAC	Mutual key	-	Counter, timestamp
**selected_nodes**	MAC	Cluster key	-	Counter
**data**	MAC	Mutual key	Encryption	Counter

## 6. Evaluation of the Proposed Protocol

In this section we evaluate the proposed protocol in detail. We compare it with its counterparts in the context of security, communication and computational complexity.

### 6.1. Security Evaluation

In this section the security comparison is done in the context of secure routing and then of secure data aggregation. We list all possible attacks for each context. We then identify each possible attack that applies in each context based on which security property (data integrity, source authentication, data confidentiality or data freshness, even availability) it attempts to compromise.

In evaluating the proposed protocol, we exclude the attacks launched by compromised nodes, or in other words internal attacks are excluded since by design the proposed protocol adopts LEACH-based secure routing and ESPDA that are not equipped to detect compromised nodes or even to punish them. Up until this stage, the proposed protocol does not employ any algorithm (such as witness-based detection to monitor a certain node’s behavior, *etc*) to punish compromised nodes. Therefore we do not consider internal attacks launched by compromised nodes.

First we evaluate the proposed protocol from the perspective of secure routing, comparing it to its counterparts (SLEACH, SecLEACH and MS-LEACH). In [4], Karlof and Wagner study the attacks and countermeasures for secure routing protocols in WSN. These attacks are:

▪ spoofed, altered, or replayed routing information;
▪ selective forwarding;
▪ sinkhole attacks;
▪ Sybil attacks;
▪ Wormholes;
▪ HELLO flood attacks;
▪ acknowledgment spoofing.

The study also presents attacks specific to LEACH-family protocols. These are selective forwarding and HELLO floods attacks. The listed attacks and their descriptions provided in [25] help our evaluation. We filter which attacks are applicable in our context. Those attacks are HELLO floods, selective forwarding, sinkhole attacks, and spoofed/altered/replaying routing information.

As we know, a successful launch of a certain attack can lead to the mounting of other attacks. For example, the HELLO floods attack launched by a malicious node may lure many other nodes to join that malicious node’s cluster, which later on results in selective forwarding or sinkhole attacks. So we can consider only the HELLO flood attack to simplify our evaluation.

Since in LEACH-based secure routing protocols, every node can take a role of either a CH or ordinary node, we consider a node impersonation attack which can be launched to target a CH or ordinary node. If we relate this attack to the previous explained HELLO flood attack, actually the HELLO flood attack is a type of CH impersonation attack since the target here is the CHs. Adversaries may impersonate any legal CH and advertise themselves to the network tricking many other ordinary nodes to join their cluster. This is similar to the HELLO floods attack. Therefore, we use the term CHs impersonation attack to represent HELLO flood attacks in our evaluation.

If the node impersonation attack’s target is ordinary nodes, then the goal of this attack is to insert false sensor readings. The significant contribution of false sensor readings can obscure the view of the actual network condition. We then add eavesdropping and an attack specific to the LEACH protocol, schedule disruption. This attack is identified when we analyze the vulnerabilities of the LEACH-based secure routing protocols in shown in Section 4.

**Figure 8:**
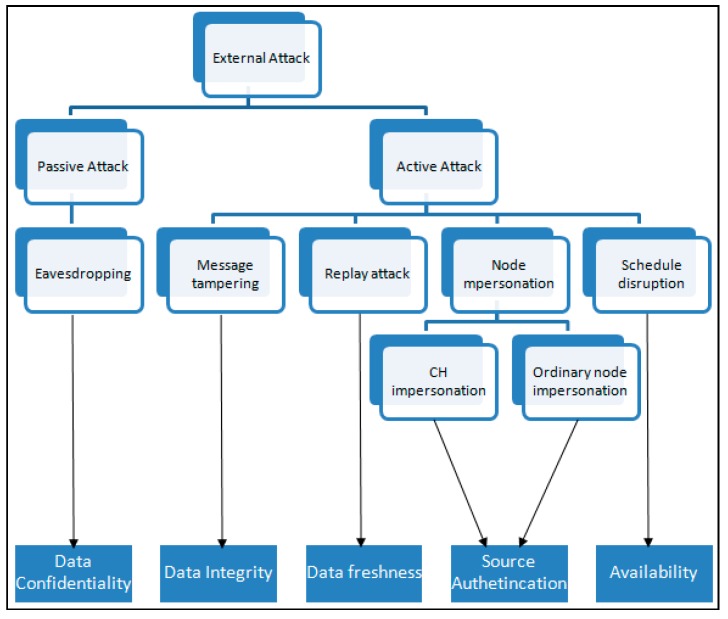
Relating each possible attack with the corresponding attacked security property.

Furthermore, we check each attack against the security objectives it attempts to compromise. In Section 3, the explained security properties are data confidentiality, data integrity, data freshness, source authentication and availability property.

Figure 8 is the diagram that summarizes our approach in the evaluation. We relate each attack with the targeted security property. By identifying each attack based on the security property it attempts to compromise, we can simply recognize how each protocol addresses the listed possible attacks. Readers may refer to Table 2 that presents the summary of countermeasures we use for our protocol to fulfill the security requirements.

Table 3 summarizes our evaluation on the list of possible attacks against SLEACH, SecLEACH, MS-LEACH and the proposed protocol from secure routing point of view. Reader may refer to the Section 3.3 for the explanation.

**Table 3:** Possible attacks addressed, in comparison with other secure routing protocols.

Possible Attacks Observed	SLEACH	SecLEACH	MS-LEACH	Proposed Protocol
Eavesdropping	No	No	Yes	Yes
Message tampering	No	No	Yes	Yes
Replay	Yes	Yes	Yes	Yes
Ordinary nodes impersonation	No	Yes	No	Yes
CHs impersonation	Yes	No	Yes	Yes
Schedule disruption	No	No	Yes	Yes

The first attack under observation is the passive attack, eavesdropping. In this attack, an adversary with a strong receiver can eavesdrop and intercept the transmitted packets. SLEACH does not prevent this attack since it does not provide confidentiality services, especially for the sensor readings and the aggregated result. SecLEACH also poses the same problem. In the other hand, MS-LEACH uses encryption to protect those data, and even the schedule messages are encrypted as well. The proposed protocol provides encrypted transmission for the sensitive data. Hence eavesdropping can be tackled.

The second attack is the message tampering. It attacks the data integrity properties. SLEACH suffers from this attack. This attack can target the join request message, the schedule message or the sensor reading transmissions of SLEACH. This is because its join request and schedule message transmission do not employ data integrity protection algorithms. In the case of its sensor reading transmissions, the transmissions are guarded by the keyed MAC using the key shared between the cluster member node and the BS, and furthermore, the sensor reading is not the input of the MAC computation. This allows adversaries to tamper with the message within cluster transmission, especially the sensor reading part, without being detected by the CH. If we observe the performance of SecLEACH against this attack, then SecLEACH also allows message tampering to take place on advertisement messages and schedule messages. MS-LEACH and the proposed protocol prevent this attack from happening. MS-LEACH uses MAC in every transmission to guarantee data integrity. The proposed protocol uses digital signatures or MAC to guarantee the data integrity of each message. Readers may refer to Table 2.

The third is the replay attack. In facing replay attacks, all these protocols are equipped with countermeasures. Though the approaches are different, all can prevent replay attacks.

As for the next two attacks, they are both node impersonation attacks but with different targeted nodes. The ordinary nodes impersonation means the adversary impersonates ordinary nodes using any valid identity while the CHs impersonation means the targeted nodes are the CHs.

In the case of ordinary nodes impersonation, since SLEACH does not authenticate any node that joins the network, adversaries can insert intruder nodes to the network by taking any valid node ids (a problem caused by unauthenticated join request messages). These intruder nodes are able to insert false readings into the aggregated data that later on will be detected by the BS. The BS drops any aggregated data which contains data from unauthenticated nodes. Even though MS-LEACH also suffers from this attack, the best thing those intruder nodes can do is just to crowd the time slots since MS-LEACH has a pairwise key mechanism, while the pair-wise key mechanism in SecLEACH prevents the insertion of these intruder nodes into the network. Using the non-interactive identity-based key agreement we provide the pair-wise key mechanism to the proposed protocol.

However, the impact would be worse if this attack is launched to impersonate CHs. SecLEACH allows CHs impersonation to take place since the advertisement messages are not authenticated. In SLEACH and MS-LEACH, the BS verifies the authenticity of the claimed CHs on behalf of the network. The proposed protocol inserts a digital signature into the advertisement messages to provide source authentication. An adversary node with strong radio power transmission may lure a lot of nodes to join its cluster. This attack can lead to many other types of attacks such as selective forwarding and sinkhole attacks.

For LEACH-based protocols, the security aspect of the schedule message should be considered. The schedule message contains the TDMA schedule for nodes within a certain cluster to take turns in reporting their sensor readings. SLEACH and SecLEACH do not facilitate source authentication services to this message distribution. They allow any intruder to disrupt the transmission schedule in any cluster. The security analysis of Section 3.3.1 and Section 3.3.2 describes how it happens. MS-LEACH and the proposed protocol provide source authentication service for this message distribution via keyed MAC. To generate MAC MS-LEACH uses a pair-wise key while ours uses a cluster key.

Therefore, in comparison with the other LEACH-based secure routing protocols, the proposed protocol can address all possible attacks under observation. The proposed protocol is really in tight competition with MS-LEACH. MS-LEACH fails to provide authenticated join request messages though it has a pairwise key mechanism that can help to achieve this, while the proposed protocol provides authenticated join request messages that block illegitimate nodes from joining the network. Therefore justifying carefully the security requirements for every involved message in the protocol is important.

The next evaluation is done on any possible attacks that attempt to hinder the proper data aggregation process. Here we compare the proposed protocol and its counterpart, ESPDA. The summary is presented in Table 4. Here we consider four attacks: eavesdropping, replay attack, node impersonation for specific CHs impersonation and message tampering.

Both protocols provide message confidentiality service for the actual data and aggregated data transmission. Therefore they address eavesdropping attacks.

**Table 4:** Possible attacks addressed, in comparison with other secure data aggregation protocols.

Possible Attacks Observed	ESPDA	Proposed Protocol
Eavesdropping	Yes	Yes
Replay	No	Yes
CHs impersonation	No	Yes
Message tampering	No	Yes

The data aggregation process can be disrupted via replay attacks. In ESPDA, the distribution of $r_{b}$, pattern seed and permutation seed are encrypted using a static network-wide key. Therefore ESPDA allows replay attacks to take place. The proposed protocol prevents this attack using a counter mechanism. We include a cluster counters into the MAC computation of those data distributions.

In ESPDA, a cluster member does not authenticate its CH (ESPDA does not provide a cluster formation description) since every node only shares a pairwise key with the BS. Any adversary can impersonate CHs and then launch other types of attack such as selective forwarding or sinkhole attacks. But one type of attack which is of concern in many secure data aggregation protocols is the stealth attack. In this attack the malicious nodes, especially the CHs, insert false sensor readings tricking the BS into accepting aggregated results which are significantly different from the actual data. As mentioned earlier our evaluation does not include attacks launched by compromised nodes, so here we consider only stealth attacks launched by inserted intruder nodes. Since the proposed protocol provides authenticated advertisement messages using digital signatures, we guarantee that the CHs are legitimate nodes and therefore the proposed protocol can address these attacks.

Another attack we observed is message tampering attacks. This attack can disrupt data aggregation in ESPDA, particularly when its target is the pattern code messages. ESPDA does not provide any data integrity service for this message, while the proposed protocol provides it by using keyed MAC.

Therefore, from Table 4 we can see that ESPDA fails to provide protection against all possible attacks observed but eavesdropping. ESPDA does not employ any countermeasure that can provide data freshness service. Its lack of pairwise key providence among sensor nodes invites CH impersonation attacks and message tampering to take place. These attacks can affect the data aggregation process outcome in very bad way since the BS may receive false reports of the network, while in the proposed protocol, the role of the pair-wise key that is affordable using identity-based key agreement and the counter mechanism gives great contribution in dealing with those attacks.

### 6.2. Communication and Computational Complexity Evaluation

This section presents an evaluation on the communication and computational cost of the proposed protocol. In order to compare our protocol with others, we make some assumptions for message sizes and computational cost.

First we provide our assumption for the length of message component. Table 5 gives the length assumption in bits for every message component. We also assume the length of the cluster key, sensor reading, pattern seed and mapping permutation to be 64 bits. That is for an approximate calculation of the encrypted data size, which is equal to the input data. The size can be varied depending on the WSN application.

**Table 5:** Assumption for the length of message components.

Message Component	Length (in bits)	Description
|id|	16	Identity of node
|nonce|	64	Random number
|sign|	480	Digital signature
|MAC|	160	MAC
|r|	64	Key identifier/index
|TDMA|	64	TDMA message size
|pc|	16	Pattern code
|T|	32	Timestamp

Table 6 and Table 7 detail the approximate size of various message types involved in the setup phase and data aggregation phase for the protocols under study. Some messages are variable-sized, some are fixed. The variable n and $n_{s}$ represent the number of cluster member and selected members whose pattern codes get selected, respectively.

Table 6 details the message size comparison for our protocol and other secure routing protocol. We also include LEACH protocol here since all other protocols have their roots in LEACH. We only consider the cluster formation phase in which the routing paths get established. Since SLEACH and MS-LEACH have extra transmissions for the legitimate CHs list and group key (steps 1.2 and 1.3 of Figure 2 and Figure 4), we add the message size for them. We assume the number of legitimate CHs for every round to be 10 CHs. The size of the message that contains the information of this list then can be calculated as 10 times the size of the id plus the MAC size, 320 bit, while the size of the message that contains group key information is 64 bits. Table 6 provides clear calculations for other message types.

**Table 6:** Message size comparison for setup phase.

Message Type	The Proposed Protocol (in bits)	SLEACH (in bits)	SecLEACH (in bits)	MS-LEACH (in bits)	LEACH (in bits)
adv ^a^	|id|+|nonce|+|sign|≅560	|id|+|MAC|≅176	|id|+|nonce|≅80	|id|+|MAC|≅176	|id|≅16
join_req	2|id|+|MAC|≅192	2|id|≅32	2|id|+|r|+|MAC|≅256	2|id|≅32	2|id|≅32
sched ^b^	|id|+|TDMA|+|enc|+|MAC|≅368	|id|+|TDMA|≅80	|id|+|TDMA|≅80	|enc|+|MAC|≅224	|id|+|TDMA|≅80
CK_req ^c^	(n+1)|id|+|MAC|≅16n+176	NA	NA	NA	NA
CK_rep ^c^	(n+2)|id|+|T|+(n+1)|enc|+|sign|≅80n+608				
Legitimate_List	NA	320	NA	320	NA
Group_key	NA	64	NA	64	NA

^a^ **sec_adv** in SLEACH and MS-LEACH; ^b^ **sched_seed_permut** in the proposed protocol; ^c^ new message type introduced in our protocol; NA = Not Applicable

Table 7 details the message size comparison involved in data aggregation phase. Therefore only our protocol and ESPDA get compared in this study. Since the transmission uses wireless medium, the unicast transmission is actually a local broadcast transmission. Therefore we consider either unicast or broadcast as one unit transmission. We also consider the wake up state where nodes do not turn off their radios. This state takes place in the setup phase of the secure routing protocols and in the ESPDA protocol (no TDMA used). Therefore, if a node sends something via unicast or broadcast transmission, the other member nodes still receive the message even though further process may not take place. We count it as receiving cost.

Based on these assumptions, we calculate the communication cost for each protocol by calculating how many bits of message get processed in each transmission. Radio energy consumption is proportional to the number of conveyed bits.

Table 8, Table 9, Table 10, Table 11, Table 12 and Table 13 provide details of the calculation for the communication costs in number of bits involved. They are calculated based on the number of transmission of each protocol shown in Figure 1, Figure 2, Figure 3, Figure 4, Figure 5 and Figure 7. Each step number in the tables corresponds to each protocol step number of the Figures. We differentiate the communication cost of CHs and ordinary nodes into transmitting and receiving cost (Tx and Rx).

**Table 7:** Message size comparison for data aggregation phase.

Message Type	The Proposed Protocol (in bits)	ESPDA (in bits)
**pattern_code**	2|id|+|pc|+|T|+|MAC|=240	|id|+|pc|+|T|=64
**selected_nodes**	$\left(n_{s}+1\right)\left|id\right|+\left|MAC\right|=16n_{s}+176$	$2\left|id\right|=32^{\;a}$
**data(within cluster)**	2|*id*| + |*enc*| + |*MAC*| = 192 + |*enc*| ≅ 256	|*id*| + |*T*| + |*enc*| + |*MAC*| = 208 + |*enc*| ≅ 272
**data(from CH to BS)**	(2 +*n_s_*)|*id*| + *n_s_*|*enc*| + |*MAC*| = 16*n_s_* + 192 + *n_s_*|*enc*| ≅ 80*n_s_* + 192	2|*id*| + |*T*| + |*enc*| + |*MAC*| = 224 + |*enc*| ≅ 288

^a^ assuming the simple request message contains the CH’s id and the corresponding selected node’s id.

**Table 8:** Communication cost of LEACH in setup phase.

Step	CH		Ordinary Node	
	Tx Cost (in bits)	Rx Cost (in bits)	Tx Cost (in bits)	Rx Cost (in bits)
1	1 × **adv** = 16	—	—	1 × **adv** = 16
2	-	*n* × **join_req** = 32	1 × **join_req** = 32	(*n* − 1) × **join_req** = 32*n* − 32
3	1 × **sched** = 80	—	—	1 × **sched** = 80
**Total**	**96**	**32*n***	**32**	**32*n* + 64**

**Table 9:** Communication cost of SLEACH in setup phase.

Step	CH		Ordinary Node	
	Tx Cost (in bits)	Rx Cost (in bits)	Tx Cost (in bits)	Rx Cost (in bits)
1.1	1 × **sec_adv** = 176	—	—	1 × **sec_adv** = 176
1.2	—	1 × **Legitimate_List** = 320	—	1 × **Legitimate_List** = 320
1.3	—	1 × **Group_Key** = 64	—	1 × **Group_Key** = 64
2	—	*n* × **join_req** = 32*n*	1 × **join_req** = 32	(*n* − 1) × **join_req** = 32*n* − 32
3	1 × **sched** = 80	—	—	1 × **sched** = 80
Total	256	32*n*+ 384	32	32*n* + 608

**Table 10:** Communication cost of SecLEACH in setup phase.

Step	CH		Ordinary Node	
	Tx Cost (in bits)	Rx Cost (in bits)	Tx Cost (in bits)	Rx Cost (in bits)
1	1 × **adv** = 80	—	—	1 × **adv** = 80
2	—	*n* × **join_reg** = 256*n*	1 × **join_reg** = 256	(*n* − 1) × **join_reg** = 256*n* − 256
3	1 × **sched** = 80	—	—	1 × **sched** = 80
**Total**	**160**	**256*n***	**256**	**256*n* − 96**

**Table 11:** Communication cost of MS-LEACH in setup phase.

Step	CH		Ordinary Node	
	Tx Cost (in bits)	Rx Cost (in bits)	Tx Cost (in bits)	Rx Cost (in bits)
1	1 × **sec_adv** = 176	—	—	1 × **sec_adv** = 176
1.2	—	1 × Legitimate_List = 320	—	1 × Legitimate_List = 320
1.3	—	1 × **Group_Key** = 64	—	1 × **Group_Key** = 64
2	—	*n* × **join_req** = 32*n*	1 × **join_req** = 32	(*n* − 1) × **join_req** = 32*n* − 32
4	*n* × **sched** = 224*n*	—	—	*n* × **sched** = 224*n*
**Total**	**224*n* + 176**	**32*n* + 384**	**32**	**256*n* + 528**

**Table 12:** Communication cost of the proposed protocol in setup phase and steady state phase.

Step	CH		Ordinary Node	
	Tx Cost (in bits)	Rx Cost (in bits)	Tx Cost (in bits)	Rx Cost (in bits)
1	1 × adv = 560	—	—	1 × adv = 560
2	—	*n* × join_req = 192*n*	1 × join_req = 192	(*n* − 1) × join_req = 192*n* − 192
3	1 × CK_req = 16*n* + 176	—	—	1 × CK_req= 16*n* + 176
4	—	1 × CK_rep = 80*n* + 608	—	1 CK_rep = 80*n* + 608
6	1 × sched_seed_permut = 368	—	—	1 × sched_seed_permut = 368
**Total (setup phase)**	**16*n* + 1104**	**272*n* + 608**	**192**	**288*n* + 1520**
7	—	*n* × pattern_code = 240*n*	1 × pattern_code = 240	—
8	1 × selected_nodes = 16*n_s_* + 176	—	—	1 × selected_nodes = 16*n_s_* + 176
9 (within cluster)	—	*n_s_* × data = 256*n_s_*	1 × data = 256 if selected, otherwise 0	—
10 (from CH to BS)	1 × data = 80*n_s_* + 192	—	—	—
**Total (steady state)**	**96*n_s_* + 368**	**240*n* + 256*n_s_***	**496 if selected, otherwise 240**	**16*n_s_* + 176**

In order to simplify our study on cluster expenditure for radio transmission in each protocol, we assume that both transmitting and receiving cost are equal. Note that their energy may differ significantly for different radio instruments. For comparison in the data aggregation phase, we assume a worst case where all cluster members get selected, so $n_{s}=n$ . Since the transmission cost for ordinary node in Table 8, Table 9, Table 10, Table 11, Table 12 and Table 13 is for individual cluster members, it should be multiplied by n to capture the whole expenditure of cluster members. Therefore the cost of one cluster is cost of 1 CH plus its n member nodes. Table 14 summarizes the comparison of the total expenditure for radio transmission within a cluster.

**Table 13:** Communication cost of ESPDA in data aggregation phase.

Step	CH		Ordinary Node	
	Tx Cost (in bits)	Rx Cost (in bits)	Tx Cost (in bits)	Rx Cost (in bits)
1	—	*n* × **pattern_code** = 64*n*	1 × **pattern_code** = 64	(*n* − 1) × **pattern_code** = 64*n* − 64
3	*n_s_* × **selected_nodes** = 32*n_s_*	—	—	*n_s_* × **selected_nodes** = 32*n_s_*
5 (within cluster)	—	*n_s_* × **data** = 272*n_s_*	1 × **data** = 272 if got selected, otherwise 0	(*n_s_* − 1) × **data** = 272*n_s_* − 272 if selected, otherwise *n_s_* × **data** = 272*n_s_*
6 (from CH to BS)	*n_s_* × **data** = 288*n_s_*	—	—	*n_s_* × **data** = 288*n_s_*
**Total**	**320*n_s_***	**64*n* + 272*n_s_***	**336 if selected, otherwise 64**	**64*n* + 592*n_s_*** − **336 if selected, otherwise 64*n* + 592*n_s_*** − **64**

**Table 14:** Comparison of intra-cluster radio transmission expenditure

Phase	Protocol	Total Expenditure (in bits)
Setupphase	LEACH	32*n*^2^ + 128*n* + 96
	SLEACH	32*n*^2^ + 672*n* + 640
	SecLEACH	256*n*^2^ + 416*n* + 160
	MS-LEACH	256*n*^2^ + 816*n* + 560
	Ours	288*n*^2^ + 2000*n* + 1712
Data aggregation phase	Ours	16*n*^2^ + 1264*n* + 368
	ESPDA	656*n*^2^ + 656*n*

From Table 14 we are able to see each protocols’ radio communication cost in polynomial form. For radio communication cost, with the increasing number of member nodes, we find the following:

- SLEACH is the cheapest secure routing protocol for the setup phase. Its radio communication expenditure is not much different from LEACH’s. This is because its modified advertisement message adds a small number of bits to the transaction. Its transmission for the list of legitimate CH and group key (in step 1.2 and 1.3, Figure 2) add some more bits. However it is still the cheapest among secure routing protocols under evaluation.
- Our protocol is the most expensive for the setup phase category. The size of the join request message and cluster key reply message are the main contributors to this cost. We have designed our protocol to have multiple steady-state phases in order to mitigate this costly setup phase. However our protocol is robust against all six possible attacks as shown in Table 3. We achieve these enhanced security features with some extra communication cost.
- Though our protocol setup phase is the most expensive, its data aggregation cost is much cheaper than its baseline protocol, ESPDA. This is because we use TDMA scheduling in our protocol design. With about 40 times of $n^{2}$ lesser transmissions than ESPDA, our scheme has three more enhanced security features than ESPDA as shown in Table 4. As the number of member nodes increases, the gap between the two schemes will increase. Because the data aggregation phase is executed more often than the setup phase, our scheme has merits compared to ESPDA.

For the practical use of the LEACH-style routing schemes introduced in our paper, they need to add more steps in their data aggregation phase when they are combined with a specific data aggregation scheme such as ESPDA. This will introduce additional communication costs. By considering this fact, because the setup phase is relatively infrequent compared to the data aggregation phase, our protocol is comparable to the other LEACH-style routing schemes when they are combined with a specific data aggregation scheme.

Another evaluation is for the amount of computation of cryptographic algorithms that takes place. Those cryptographic algorithms include hash function, symmetric encryption/decryption, MAC computation, digital signature generation and verification. We denote by C(θ) the computation cost of operation θ.

We consider hash operation cost and MAC computation equal since MAC computation employs a hash function, therefore we represent each of both as one unit of hash operation, h. Since it is symmetric encryption/decryption, we consider both costs of operation equal and represent each of them as one unit of encryption operation, enc. An exception is digital signature generation and verification. Both of them do not have equal computational costs, therefore we represent the signing operation as $\sigma_{g}$ and its verification operation as $\sigma_{v}$. As here we do not strict the proposed protocol to any specific digital signature algorithm, the computation cost can be expanded according to the chosen digital signature algorithm. Table 15 presents the cryptographic computation cost.

Since we cannot tell exactly the execution time of each algorithm, we still can compare them by ordering them based on computation complexity. Let’s say $\tilde{m}$ is modular multiplication in ${\mathbb{Z}}_{q}^{*}$, E is exponentiation in G (equivalent to scalar multiplication in ECC), M is the multiplication in G (equivalent to point addition in ECC) and, previously stated, C(h) is hash operation and C(enc) is symmetric encryption/decryption. We can sort the computational complexity of the algorithms as follows: $E>M>\tilde{m}>C\left(enc\right)>C\left(h\right)$.

**Table 15:** Computational cost comparison.

Phase	Node’s Role	SLEACH	SecLEACH	MS-LEACH	ESPDA	The Proposed Protocol
Setup	CH	3C(h)	nC(h)	6C(h)+C(enc)	NA	$C\left(\sigma_{g}\right)+C\left(\sigma_{v}\right)+\left(2n+3\right)C\left(h\right)+2C\left(enc\right)$
	Ord. Node	2C(h)	C(h)	5C(h)+C(enc)		$2C\left(\sigma_{v}\right)+3C\left(h\right)+2C\left(enc\right)$
Data Aggregation	CH	NA	NA	NA	0	$\left(2n+n_{s}+3\right)C\left(h\right)$
	Ord. Node				C(h)+C(enc) (*), Otherwise **0**	4C(h)+C(enc) (*), otherwise 3C(h)

n, $n_{s}$: the amount of nodes within a certain cluster and of selected nodes, respectively; NA: Not applicable; (*): if it is selected node.

In [24], Liu *et al.* measured that their digital signing operation equals $\tilde{m}$ and the signature verification equals to 2E+M. We can tell that the verification is more costly than their signing operation. From the comparison of computational complexity shown in Table 15, it is very obvious that the proposed protocol is the most expensive one in its classes, especially in setup phase. If we refer to the Table 2 on how we fulfill the security requirements of the proposed protocol summarized in Table 1, we can see that the proposed protocol uses cryptographic approaches to meet them. This cryptographic approach is a kind of trade-off between computation cost and security level since the proposed protocol can tackle all listed possible attacks at the sacrifice of computational burden (readers may refer to Table 3 and Table 4 in the security evaluation section).

## 7. Conclusions

To the best of our knowledge, only the work of Wu *et al.* and SELDA incorporate secure routing schemes into their secure data aggregation protocols. In their protocols, the secure routing algorithms are only activated when the network nodes do not work honestly. Their secure routing protocols work on-demand or reactively. This means that there has been no work that lays secure routing protocol as the foundation for the secure data aggregation protocol. We argue that the role of a secure routing would be rendered useless if the data aggregation scheme built on it is not secure. Conversely, the secure data aggregation protocol needs a robust underlying routing protocol as its foundation in order to be effectively optimal.

In an attempt to provide a solution to this problem, we propose a secure routing protocol that considers secure data aggregation. We combine a LEACH-based security protocol with ESPDA, a secure data aggregation protocol suitable for cluster-based WSNs. The protocol design has been presented with evaluations on security, communication and computational complexity analysis. There is always trade-off between security and energy-efficiency. Minimizing the gap between them is a challenging task for WSN protocol design. Our protocol spends a lot of its energy-efficient features for facilitating security compared to the other protocols under evaluation. The point we would like to highlight is the importance of considering secure routing and secure data aggregation together in designing a protocol for WSNs in order to achieve energy-aware yet secure protocols.

## References

[1] Seema A., Reisslein M. (2011). Towards efficient wireless video sensor networks: A survey of existing node architectures and proposal for a Flexi-WVSNP design. IEEE Commun. Surv. Tutor..

[2] Tavli B., Bicakci K., Zilan R., Barcelo-Ordinas J.M. (2012). A survey of visual sensor network platforms. Multimed. Tools Appl..

[3] El-Semary A.M., Abdel-Azim M.M. (2013). New Trends in Secure Routing Protocols for Wireless Sensor Networks. Int. J. Distributed Sens. Netw. (Hindawi).

[4] Karlof C., Wagner D. (2003). Secure Routing in Wireless Sensor Networks: Attacks and Countermeasures. Ad Hoc Netw..

[5] Perrig A., Szewczyk R., Wen V., Culler D., Tygar J.D. (2000). SPINS: Security Protocols for Sensor Networks. Wirel. Netw..

[6] Ozdemir S., Yang X. (2009). Secure Data Aggregation in Wireless Sensor Networks: A Comprehensive Overview. Comput. Netw..

[7] Alzaid H., Foo E., Nieto J.G., Ljiljana B., Mirka M (2008). Secure Data Aggregation in Wireless Sensor Network: A Survey. Proceedings of the Australian Information Security Conference.

[8] Wu K., Dreef D., Sun B., Xiao Y. (2007). Secure data aggregation without persistent cryptographic operations in wireless sensor networks. Ad Hoc Netw..

[9] Ozdemir S., Ichikawa H., Cho W.-D., Sato I., Hee Y.Y. (2007). Secure and reliable data aggregation for wireless sensor networks. Ubiquitous Computing System.

[10] Heinzelman W.R., Chandrakasan A., Balakrishnan H. Energy-efficient communication protocol for wireless microsensor networks. Proceedings of the 33rd Annual Hawaii International Conference on System Sciences.

[11] Triana M.R., Lee S.G., Lee H.J. Survey on LEACH-based Security Protocols. Proceedings of 16th International Conference on Advanced Communications Technology (ICACT 2014).

[12] Triana M.R., Lee S.G., Lee H.J. Secure Tree Construction for the Secure Hop-by-Hop Data Aggregation Protocol for Wireless Sensor Networks. Proceedings of the 9th Asia Pacific International Conference on Information Science and Technology (APIC-IST 2014).

[13] Ferreira A.C., Vilaca M.A., Oliveira L.B., Habib E., Wong H.C., Loureiro A.A.F., Pascal L., Dini P. (2005). On the security of cluster-based communication protocols for wireless sensor networks. Networking-ICN 2005.

[14] Oliveira L.B., Wong H.C., Bern M., Dahab R., Loureiro A.A.F. SecLEACH—A Random Key Distribution Solution for Securing Clustered Sensor Networks. Proceedings of the 5th IEEE International Symposium on Network Computing and Applications.

[15] Wang J., Yang G., Chen S., Sun Y. Secure LEACH routing protocol based on low-power cluster-head selection algorithm for wireless sensor networks. Proceedings of the International Symposium on Intelligent Signal Processing and Communication Systems.

[16] Abuhelaleh M.A., Mismar T.M., Abuzneid A.A. Armor-LEACH-energy efficient, secure wireless networks communication. Proceedings of the IEEE 17th International Conference on Computer Communications and Networks.

[17] El-Saadawy M., Shaaban E. Enhancing S-LEACH security for wireless sensor networks. Proceedings of the IEEE International Conference on Electro/Information Technology.

[18] Eschenauer L., Gligor V.D. A key-management scheme for distributed sensor networks. Proceedings of the 9th ACM conference on Computer and communications security.

[19] Çam H., Ozdemir S., Nair P., Muthuavinashiappan D., Sanli H.O. (2006). Energy-efficient and secure pattern based data aggregation for wireless sensor networks. Comput. Commun..

[20] Shamir A. Identity-Based Cryptosystems and Signature Schemes. Proceedings of CRYPTO 1984, Springer-Verlag: 1984, LNCS 196.

[21] Boneh D., Franklin M. Identity-Based Encryption from Weil Pairing. Proceedings of CRYPTO 2001, Springer-Verlag 2001, LNCS 2139.

[22] Oliveira L.B., Aranha D.F., Gouvea C.P., Scott M., Camara D.F., Lopez J., Dahab R. (2011). TinyPBC: Pairings for authenticated identity-based non-interactive key distribution in sensor networks. Comput. Commun..

[23] Sakai R., Ohgishi K., Kasahara M. Cryptosystems based on pairing. Proceedings of the SCIS2000-C20.

[24] Liu J., Baek J., Zhou J., Yang Y., Wong J.W. (2010). Efficient online/offline identity-based signature for wireless sensor network. Int. J. Inf. Secur..

[25] Akyildiz I.F., Su W., Sankarasubramaniam Y., Cayirci E. (2002). Wireless sensor networks: A survey. Comput. Netw..

